# Diabetes and bone

**DOI:** 10.20945/2359-3997000000552

**Published:** 2022-11-10

**Authors:** Iana Mizumukai de Araújo, Mariana Lima Mascarenhas Moreira, Francisco José Albuquerque de Paula

**Affiliations:** 1 Universidade de São Paulo Faculdade de Medicina de Ribeirão Preto Departamento de Clínica Médica Ribeirão Preto SP Brasil Departamento de Clínica Médica, Faculdade de Medicina de Ribeirão Preto, Universidade de São Paulo, Ribeirão Preto, SP, Brasil

**Keywords:** Glucose, fracture, insulin, FRAX, diagnosis

## Abstract

Globally, one in 11 adults has diabetes mellitus of which 90% have type 2 diabetes. The numbers for osteoporosis are no less staggering: 1 in 3 women has a fracture after menopause, and the same is true for 1 in 5 men after the age of 50 years. Aging is associated with several physiological changes that cause insulin resistance and impaired insulin secretion, which in turn lead to hyperglycemia. The negative balance between bone resorption and formation is a natural process that appears after the fourth decade of life and lasts for the following decades, eroding the bone structure and increasing the risk of fractures. Not incidentally, it has been acknowledged that diabetes mellitus, regardless of whether type 1 or 2, is associated with an increased risk of fracture. The nuances that differentiate bone damage in the two main forms of diabetes are part of the intrinsic heterogeneity of diabetes, which is enhanced when associated with a condition as complex as osteoporosis. This narrative review addresses the main parameters related to the increased risk of fractures in individuals with diabetes, and the mutual factors affecting the treatment of diabetes mellitus and osteoporosis. Arch Endocrinol Metab. 2022;66(5):633-41

## INTRODUCTION

Diabetes mellitus (DM) and osteoporosis are two of the most important diseases that affect human beings, overburden countries’ healthcare systems, result in high costs, and reduce life expectancy. Moreover, both diseases affect the quality of life, with DM leading to cardiovascular, ocular, renal, and neural damage and osteoporosis robbing its patients of their autonomy and mobility.

Multiple pieces of evidence indicate that not only muscle and adipose tissue contribute to the regulation of energy metabolism but that peptides produced by bone cells are also part of the orchestration that involves the metabolic control of glucose, lipids, and proteins (1–3). Similarly, several hormones are important for the regulation of energy metabolism and influence bone remodeling (e.g., insulin and epinephrine). As expected, both type 1 DM (T1DM) and type 2 DM (T2DM) are associated with an increased risk of fracture. The mechanisms involved in the deterioration of bone strength are not yet clearly elucidated, but the loss of bone density is certainly not the main determinant of fracture susceptibility in DM. This aspect is more evident in T2DM, where bone density is preserved or even increased, but it is also observed in T1DM, where the increase in fracture risk is much greater than the loss of bone density would suggest (4). Experimental and clinical research on the mechanisms and various diagnostic and treatment aspects of osteoporosis in diabetes has incited great interest. However, in clinical practice, there is a gap in the diagnosis and preventive treatment of osteoporosis in patients with DM. Therefore, this narrative review aims to provide an up-to-date overview of the primary aspects that link osteoporosis to DM, including the effects of DM treatment drugs that increase the risk of fracture and the possible diabetogenic effects of drugs used to treat osteoporosis.

### Bone density in type 1 diabetes mellitus

The introduction of insulin therapy and improvement of insulin replacement in patients with T1DM have mitigated the emergence of microvascular complications and allowed an increase in this population’s life expectancy. However, with the increasing number of studies on the comorbidities of diabetes, it has been acknowledged that T1DM increases the risk of fractures (5,6). The relative risk for all fractures in T1DM is 3.16 (95% confidence interval [CI] 1.51-6.63; P = 0.002) according to a meta-analysis performed by Shah and cols. (7). *Women still are four times and men two times more likely to have fractures when diagnosed with T1DM* (7).

Studies remain contradictory regarding the bone density of individuals with T1DM, and there appear to be sex differences. A prospective study followed-up men and women with T1DM for 5 years and observed that bone density did not change in women during that period, but there was a reduction in bone density in the femoral neck of men (8). In addition, a meta-analysis reported a slight reduction in bone density only in the femoral neck of adults with T1DM while it remained stable in the lumbar spine (9).

The traditional lean phenotype of T1DM is being changed by the combination of excess calorie intake and compensation with high insulin doses. This combination leads to weight gain, with changes in body composition and fat accumulation, which may increase the risk of developing obesity-related diseases (10).

Body weight has a positive relationship with bone density (11). Thus, recent studies have observed that individuals with T1DM with normal or increased weight have bone density similar to normoglycemic individuals (12,13). Therefore, as in T2DM, the increased risk of fractures in T1DM may be related to a decrease in bone quality. This hypothesis is supported by several studies showing that the trabecular bone score (TBS), an indirect measure of trabecular bone microarchitecture, is lower in T1DM than in a control group (14,15).

### Bone density in type 2 diabetes mellitus

Regardless of sample size, studies show that T2DM does not negatively affect bone density, even when compared with a group of similar body weight (16,17). Later prospective studies clearly showed that although T2DM does not result in specific loss of bone density, it is associated with a higher risk of fracture (18,19). Individuals with diabetes are 32% more likely to have a fracture compared to those without diabetes (20). *The Rotterdam study evaluated the* bone density *of 792 subjects, divided into control and T2DM groups. The results clearly showed a greater* bone density *in the T2DM group than in the control group. However, the same individuals were followed-up for 6.8 years, and it was observed that the T2DM group, regardless of sex, had a higher frequency of fractures than the control group* (21). Thus, it can be concluded that the densitometry test alone underestimates the risk of fractures in individuals with T2DM.

Insulin resistance is clearly related to the emergence of cardiovascular diseases and T2DM. However, a recent study found that there is no relationship between bone density and the various parameters related to insulin resistance (22). On the other hand, insulin resistance in osteoblasts may decrease osteoblastic activity, as observed in a mice model (23). A low rate of bone remodeling is common in several conditions associated with insulin resistance (24,25)

### Structural and metabolic bone changes in type 2 diabetes mellitus

#### Bone remodeling

Osteocalcin is considered a biochemical marker of bone remodeling and is involved in several physiological processes, such as the maintenance of normal bone mineralization and deceleration of growth-cartilage mineralization. A hallmark of bone disease in T2DM is decreased bone formation, biochemically translated as a reduction in serum osteocalcin (17). In a recent study, not only was it found that the production of osteocalcin was impaired but also that serum levels of osteocalcin are negatively associated with bone density. These data suggest that low bone remodeling activity is a determining factor in maintaining bone density in T2DM (17). These data have been replicated in the literature. A previous study reported that individuals with diabetes had reduced osteocalcin when compared to a control group, and serum levels of osteocalcin were also negatively associated with those of glucose and insulin as well as with the Homeostatic Model Assessment for Insulin Resistance (HOMA-IR) score (26). In hyperglycemic conditions, the Wnt/β-catenin pathway is suppressed, thereby modulating bone formation owing to the increase of its inhibitors Dickkopf-related protein 1 (DKK1) and sclerostin (27). An increase in DKK1 and sclerostin was observed in children with T1DM, and this increase was correlated to hyperglycemia (28).

### Advanced glycation endproducts and ferroptosis

The accumulation of advanced glycation endproducts (AGEs) in bone possibly decreases bone elasticity by structural modification of collagen, altering the biomechanical properties of bone (29). *In vitro* studies indicate that pentosidine decreases osteoclast differentiation and activity (30). AGEs appear to suppress cell differentiation of both osteoblasts and osteoclasts in a dose-dependent manner (31). One study found a relationship between increased pentosidine levels and an increased incidence of clinical fractures in individuals with diabetes (relative risk [RR] 1.42; 95% CI) (32).

Ferroptosis has recently been recognized as a cell death programming mechanism induced by an increased production of reactive oxygen species and iron-dependent lipid peroxidation. In a recent study, Yang and cols. found that the metabolic environment of DM is favorable to lipid peroxidation, iron overload, and activation of ferroptosis. Additionally, their study showed that inhibition of this process can rescue osteocytes. Therefore, these authors not only point to a new mechanism for the emergence of bone damage in diabetes but also to a potential treatment path (33).

### Bone marrow adipose tissue

Studies of bone marrow adiposity in T2DM remain controversial. A recent study evaluated 78 individuals, divided into a control group and groups for patients with obesity and diabetes. Bone marrow adiposity was similar between the groups, and a trend toward a negative association between lumbar-spine bone density and bone marrow adiposity was noted (17). On the other hand, another study reported that women with T2DM have higher levels of saturated lipids and lower levels of unsaturated lipids in their bone marrow than women without diabetes (34). Moreover, a previous study found that saturated lipids are associated with an increased risk of fracture in T2DM (34).

Osteoblasts and adipocytes originate from the same mesenchymal stem cells (24). It is possible that an increase in adipocyte differentiation may occur to the detriment of the osteoblast lineage. An expanded bone marrow adipose tissue may produce inflammatory cytokines, promoting osteoclastogenesis. Furthermore, in mice with streptozotocin-induced T1DM, an increase in osteoclastogenesis was observed, related to an increased expression of the receptor activator of nuclear factor kappa-Β ligand (RANKL) protein in bone marrow adipocytes (35).

### Bone microarchitecture

**Figure 1:**
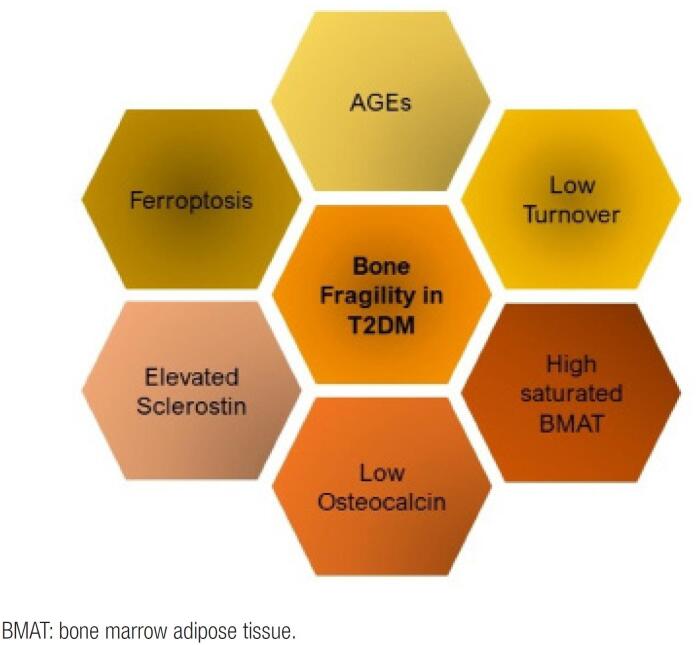
Mechanisms involved in bone fragility in T2DM. AGEs: advanced glycosylation end products.

High-resolution peripheral quantitative computed tomography (HR-pQCT) can quantitatively detail cortical and trabecular bone parameters. The results obtained by HR-pQCT performed in individuals with T2DM suggest the occurrence of intracortical porosity in parallel with an increase in trabecular volume (36). In individuals with T1DM and microvascular disease, significant deficits in cortical and trabecular bone microarchitecture parameters appear to occur in the distal radius and tibia compared to control subjects (37). One study using the microindentation technique evaluated individuals with and without diabetes (38). The bone material strength index (BMSi) was impaired in postmenopausal women with diabetes. Another study not only confirmed the BMSi data but also observed a negative correlation with blood glucose (39). The texture pattern of bone can be evaluated by the TBS, which is an estimate based on the variations of gray levels in the pixels of images of the lumbar spine from bone densitometry examinations. A previous study found a reduction in the TBS in T2DM and reported that TBS values were higher in individuals with better glycemic control (40). Figure 1 shows the mechanisms involved in bone fragility.

### Medications

#### Insulin

Insulin resistance does not seem to be a detrimental factor for bone density from a quantitative point of view, and the literature shows evidence of an increase in bone mineral density at all sites in patients with T2DM (22,41). Lipodystrophic syndrome (LDS) can be considered a natural model of hyperinsulinemia, and studies have shown that bone mineral density is preserved in both partial familial forms (42) and generalized congenital forms (43) of LDS. However, several studies have reported that the use of insulin is associated with an increase in fractures, with an approximately 38% higher risk among patients using insulin than those using oral antidiabetics (44). Furthermore, in a meta-analysis with 138,690 individuals (45), insulin use was positively associated with fracture risk (RR = 1.24, 95% CI 1.07-1.44; P = 0.004). This higher risk can be explained by episodes of hypoglycemia, and the consequent occurrence of falls (46). It must be noted that the type of insulin can influence this scenario. Insulin analogs such as glargine have a lower risk of hypoglycemia and a lower association with risk of fractures than neutral protamine Hagedorn insulin (47). Notably, other factors can interfere in the relationship between insulin use and fractures, such as the duration of the disease and coexistence of complications, especially diabetic microvascular disease, retinopathy, and neuropathy (48).

#### Metformin

Biguanides have been used for over seven decades, and metformin is considered a first-line treatment for T2DM. However, its role in bone metabolism remains controversial. Some preclinical studies have demonstrated a positive *in vitro* action of this medication on the differentiation and mineralization of osteoblastic cells through the activation of the adenosine monophosphate (AMP)-activated protein kinase (AMPK) pathway (49). Metformin also has an osteogenic effect on the recruitment of bone marrow progenitor cells for differentiation into osteoblasts, mediated by the activation of AMPK and the runt-related transcription factor (Runx2), both *in vitro* and *in vivo* (50). Conversely, in a study with *in vivo* murine models, no effect of metformin use on bone density or fracture healing was observed (51). Some clinical studies reported that metformin use was associated with reduced risk of fractures (hazard ratio [HR] 0.81; 95% CI 0.70-0.93) (19). These results were not confirmed in other studies, which did not observe a reduction in the risk of fractures in postmenopausal women or in men (52,53). Therefore, the effect of metformin appears to be either positive or neutral in relation to bone density and fracture risk, making it safe for use in patients with osteoporosis.

#### Sulfonylureas

Sulfonylureas are a class of insulin secretagogue medications. Regarding bone remodeling markers, they seem to have a neutral or reducing effect on C-terminal (CTX) and N-terminal telopeptides of type I collagen (53,54). As for the risk of fractures, a meta-analysis with 11 studies involving more than 200,000 individuals showed that the risk of having a fracture in individuals using sulfonylureas was 1.14 (95% CI 1.08-1.19), similar to the use of thiazolidinediones (TDZs), higher than for metformin, but lower than for insulin (55). Therefore, in view of the increased risk of hypoglycemia associated with this class of drugs, their use should be avoided in individuals at high risk of fracture.

#### Incretin mimetics

Incretin mimetics include dipeptidyl-peptidase 4 inhibitors (iDPP4) and glucagon-like peptide (GLP1) analogs. Their role in bone metabolism appears to be related to the differentiation of mesenchymal cells into osteoblasts, which express receptors for GLP1 and glucose-dependent insulinotropic peptide (GIP) (56). However, available data from human studies are not conclusive. A meta-analysis of more than 9,000 patients using iDPP4 compared to placebo (57) showed a reduced risk of fractures (odds ratio [OR] = 0.90, 95% CI 0.37-0.99), a finding that was not confirmed in another meta-analysis of observational real-life studies (58). Regarding GLP1, one systematic review (59) including 38 studies found protection against all fractures (OR = 0.71, 95% CI 0.56-0.91). Another meta-analysis only found reduced risk of hip fractures in the GLP1 subgroup (58). Therefore, these medications do not appear to have detrimental effects on bone density.

#### Sodium-glucose cotransporter 2 (SGLT2) inhibitors

Sodium-glucose cotransporter 2 (SGLT2i) inhibitors are a group of medications that show a wide range of effects beyond glycemic control, with benefits in cardiovascular outcomes and diabetic kidney disease. Initially, there were concerns regarding bone health, as the CANVAS study associated the use of canagliflozin with reduced bone mineral density in the hip (60) and an increased risk of extremity fractures (61). The CREDENCE trial evaluated, as a secondary analysis, the incidence of bone fracture in T2DM submitted to canagliflozin therapy, but showing impairment in kidney function. In this study, no difference was found in occurrence of fractures between the canagliflozin and placebo groups. As such, further studies will be necessary to clearly define the skeletal effects of canagliflozin (62). In subsequent meta-analyses and systematic reviews involving canagliflozin, dapagliflozin and empagliflozin, as well as real-world evidence in the literature, no increased risk of fractures was observed (58,63,64).

### Thiazolidinediones

Thiazolidinediones are peroxisome proliferator-activated receptor gamma (PPARγ) agonists. They improve insulin sensitivity, act on the redistribution of visceral adipose tissue to the subcutaneous tissue, and reduce lipotoxicity (65,66). In bone marrow adipose tissue, PPARγ stimulates the differentiation of mesenchymal stem cells into adipocytes impeding the osteoblast formation pathway. Medications (such as TZDs) or stimuli that favor the activation of PPARγ impair bone formation, leading to reduced bone mineral density and predisposition to fractures (56). In the literature review, an increased risk of fractures was observed in women (OR = 1.94; 95% CI 1.60-2.35; P < 0.001) but not in men (OR = 1.02; 95% CI 0.83-1.27; P = 0.83), and this risk was similar with both rosiglitazone and pioglitazone. In addition, the use of TZDs was also associated with a reduction in bone mineral density in the lumbar spine, total hip, and femoral neck, and a correlation, albeit not significant, was found between the cumulative use of TZDs and risk of fractures (67). These data were reinforced by a meta-analysis study, confirming the association of this class with an increased risk of fractures (68). In view of this evidence, the use of TZDs should be avoided in postmenopausal women owing to the increased risk of fractures.

### Clinical management of osteoporotic patients with diabetes

In 2018, the International Osteoporosis Foundation (IOF) published recommendations for the diagnosis and management of bone fragility in diabetes (69). The criteria for the diagnosis and treatment of osteoporosis in patients with diabetes are the same as for the general population: the presence of fragility, osteoporotic fractures, or low bone mineral density in postmenopausal women. However, as mentioned above, a significant proportion of individuals with T2DM have normal or increased bone density, and the risk of fracture is underestimated by densitometry. To improve fracture risk estimation, the IOF proposed several adaptations to the fracture risk analysis in patients with T2DM.

#### Dual-energy X-ray absorptiometry (DXA)

**Figure 2:**
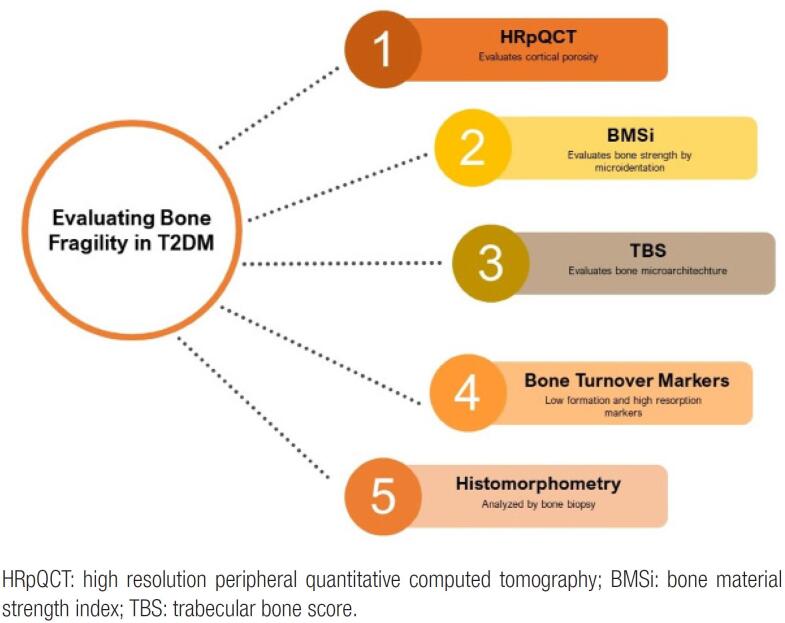
Different options to evaluate bone microstructure (HRpQCT, bone histomorphometry and TBS), strength (BMSi) and metabolism (biochemical markers).

Specifically for patients with diabetes, the IOF suggests to reduce the threshold of therapeutic intervention to a T-score of ≤−2.0 standard deviations (SD), instead of −2.5 SD as recommended for the general population. This suggestion is based on data showing that low bone density is a risk factor for fractures in T2DM and occurs at a higher T-score level than in the non-diabetic population. Densitometric evaluation should start at age 50 or 5 years after the diagnosis of T2DM in the absence of other risk factors, and it should be repeated every 2 or 3 years, as needed by each patient (69). Owing to the higher risk of vertebral fractures in this population (70), evaluation of subclinical fractures by spine radiographs or by a vertebral fracture assessment is recommended. Recently, a group of experts suggested the incorporation of TBS into the FRAX algorithm to refine the analysis of fracture risk in T2DM (71). Figure 2 shows different options for the evaluation of bone structure, strength and metabolism in T2DM.

#### World Health Organization fracture-risk algorithm (FRAX^®^)

The FRAX risk stratification calculator includes T1DM as a risk factor for secondary osteoporosis in patients aged > 40 years. However, there are no parameters that include the increased risk inherent to T2DM, and again, the risk of fracture is underestimated in these patients. In this case, there are several suggestions in the literature for adjusting FRAX. Leslie and cols. suggested four alternatives: 1. select rheumatoid arthritis (RA) in the risk factors; 2. reduce the informed femoral neck T-score by 0.5 SD; 3. increase the patient’s age by 10 years; and 4. correct FRAX with the TBS if available (72). The IOF recommends choosing one of these adjustments for FRAX and suggests using the RA option in the risk factors as equivalent to T2DM.

#### Non-pharmacological treatment

There are no specific nutritional recommendations for patients with T2DM and osteoporosis. The Brazilian guidelines for the treatment of osteoporosis recommend men and women aged > 50 years to consume 1,200 mg of calcium a day, preferably through milk and dairy products or supplements, and the use of maintenance doses of vitamin D, 1,000-2,000 IU daily, targeting 30 ng/mL (73). In the context of diabetes, hypoglycemia is an adverse event that must be avoided due to the risk of falls. In frail or elderly patients, strict control of diabetes is not recommended. Regular aerobic physical activity (such as walking) and muscle strengthening are indicated both to improve glycemic control and to prevent sarcopenia and falls (74,75). In addition, medications that reduce bone density, such as glitazones, should be replaced or discontinued, and alcohol consumption and smoking should be discouraged.

#### Pharmacological treatment

Several lines of evidence suggest that bone cells have endocrine activity, modulating energy metabolism (e.g., through osteocalcin and lipocalin) (76). Therefore, interference in bone remodeling could potentially affect glucose metabolism (77). However, few studies have addressed whether anti-osteoporosis medications have any impact on glucose metabolism.

There is no consensus in the literature regarding the effects of bisphosphonates (BPPs) on blood glucose. A large retrospective study conducted in the United Kingdom showed a reduced risk of diabetes with the use of BPPs (78), and some prospective studies showed improvement in fasting glucose and hemoglobin A1c (HbA1c) as well as a reduced risk of T2DM (79,80). However, these findings are not described in the pivotal studies of alendronate and zoledronic acid (the FIT and HORIZON studies, respectively), which did not find any differences in fasting glucose values or in the incidence of diabetes (81).

RANKL may reduce glucose tolerance. Therefore, denosumab, an anti-RANKL antibody, could have some beneficial effect on insulin sensitivity (82). However, no significant beneficial effect of denosumab on fasting blood glucose or on the incidence of diabetes was found in the FREEDOM trial (81). Additionally, in another study, the administration of denosumab to 48 postmenopausal women with osteoporosis did not change HOMA-IR or fasting glucose after 24 weeks, although there was a decrease in CTX and osteocalcin (83).

Primary hyperparathyroidism is associated with hyperglycemia. Therefore, there was concern about the effects of parathyroid hormone analogs on glucose metabolism (77,84). In the case of teriparatide (TPD), these findings remain undefined. Two studies conducted by Anastasilakis’ group showed different findings. The first analyzed 25 women using TPD and 19 women with primary hyperparathyroidism before and after surgical treatment. While the former group did not show any significant changes in glucose homeostasis, the latter showed greater insulin resistance, which improved after surgical resolution (85). In the other study, 23 postmenopausal women with osteoporosis were evaluated with a 75-g oral glucose tolerance test after 6 months of TPD use. The results showed a slight, subclinical, but significant increase in glycemia in the glycemic curves (86). On the other hand, treatment with TPD was positively associated with an improvement in HbA1c in patients with glucocorticoid-induced osteoporosis (87).

Another anabolic medication available in clinical practice is the anti-sclerostin antibody romosozumab. Owing to its more recent use, there are few data in the literature on its association with glycemic metabolism. A 2022 meta-analysis (88) evaluated 5,069 patients and observed that high sclerostin levels were associated with a higher risk of diabetes (OR = 1.25, 95% CI 1.12-1.37) and high fasting glucose levels (OR = 1.15, 95% CI 1.04-1.26). Indirectly, these data are inconsistent with the findings of the ARCH study, which cast doubt on the cardiovascular safety of romosozumab. These findings were not observed in other safety studies with the same medication (89). Therefore, further studies are needed to better characterize the cardiovascular effects associated with this medication.

Thus, as there is no strong evidence of any detrimental effects of osteoporosis treatment on diabetes, the approach to osteoporotic patients with diabetes still follows the general recommendations, and BPPs remain the first-line treatment. In older patients with impaired kidney function or those using multiple oral medications, denosumab may be preferred. Finally, the use of anabolic medications remains indicated in patients with severe osteoporosis, previous fractures, or in those at very high risk of fractures (69).

In conclusion, this article emphasizes that bone fragility should be part of the investigation protocols for the chronic complications of T1DM and T2DM, particularly in individuals who have had diabetes for more than five years. While anti-osteoporosis agents do not appear to negatively impact glucose metabolism, some oral antidiabetic drugs may impair bone density maintenance. This should be considered in the therapeutic choice, particularly for postmenopausal women.
